# Carpal tunnel syndrome caused by a vascular malformation in a 48-year-old woman

**DOI:** 10.1016/j.ijscr.2020.04.089

**Published:** 2020-05-13

**Authors:** Shinsuke Morisaki, Shinji Tsuchida, Ryo Oda, Hiroyoshi Fujiwara

**Affiliations:** Department of Orthopaedics, Graduate School of Medical Science, Kyoto Prefectural University of Medicine, Kawaramachi Hirokoji, Kamigyo-ku, Kyoto 602-8566, Japan

**Keywords:** Carpal tunnel syndrome, Vascular malformation, Nerve compression

## Abstract

**Introduction:**

Vascular malformation can present as nerve compression syndrome in middle age due to slow lesion growth, after first appearing in childhood. In a case of vascular malformation involving the peripheral nerve, surgical resection may cause uncontrollable hemorrhage and further nerve compression. Therefore, a more conservative approach is needed in such cases.

**Case presentation:**

A 48-year-old woman complained of pain and paresthesia of the fingers, and was diagnosed with a large vascular malformation expanding from the proximal forearm to the palm on MRI. Because of the size of the lesion and involvement of soft tissue, resection had a risk of major hemorrhage and incomplete removal. Thus, carpal tunnel release was performed as a less invasive procedure, and was effective for reducing pain while avoiding the risks associated with resection.

**Discussion:**

Our case indicates that a vascular lesion is a possible cause of nerve compression in the carpal tunnel. Finger numbness and pain were caused by this nerve compression, but the lesion itself was not painful. The median nerve was separate from the venous structure, and the case was diagnosed as an extraneural type.

**Conclusion:**

Symptoms that develop in middle age due to an extraneural vascular malformation in the carpal tunnel can be treated successfully using carpal tunnel release, instead of more invasive resection of the lesion.

## Highlights

• Vascular malformation can present nerve compression syndrome in a middle age because of slow growth, though it appeared in childhood.
• The less invasive operative treatment of carpal tunnel release is effective to reduce pain for the large size vascular malformation.
• The carpal tunnel release has advantage of improving the complaint without damaging the tumor.

## 1 Introduction

Vascular malformation may occur in any part of the body and is difficult to diagnose and treat, especially if the condition causes peripheral nerve compression [1]. In carpal tunnel syndrome, the median nerve is compressed, causing pain and numbness in the hand. This syndrome is usually idiopathic, but a certain etiology is present in some patients. A space occupying lesion is included among the causes, but this is relatively rare [2].

Here, we report a case in which vascular malformation presenting as median nerve neuropathy was diagnosed in middle age due to slow growth of the lesion. Resection had a risk of inadequate excision and subsequent persistent expansion, whereas carpal tunnel release had the advantage of improving the symptoms without affecting the lesion, as well as being a less invasive procedure. This case is reported in line with SCARE criteria [3].

## 2 Case presentation

The patient was a 48-year-old woman who had occasionally felt pain and swelling in her left forearm since childhood, but she had not visited a clinic because the pain resolved naturally. She had no medication history or surgical history, and no relevant family history. She had felt increasing pain and numbness in her left finger for about 1 month, and she was referred to our hospital after the pain increased further.

**Fig. 1:**
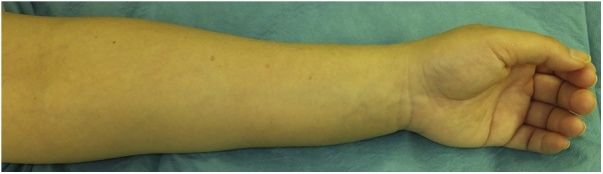
Macroscopic view of the left hand showing swelling of the volar aspects of the forearm and hand.

Her forearm were broadly distended (Fig. 1) and the circumference of the left wrist was 1.2 cm longer than that of the right wrist. Grip strength was reduced by 11.4 kg on the left. Pain in the dorsiflexion of the wrist joint was noted, and the patient complained of numbness in the fingers. Electromyography showed a decreased amplitude of motor nerve conduction of the median nerve compared to the healthy side, but there was no delay in latency. Sensory nerve conduction velocity was undetectable on the affected side in the median nerve. The visual analogue scale for pain was 30/100.

**Fig. 2:**
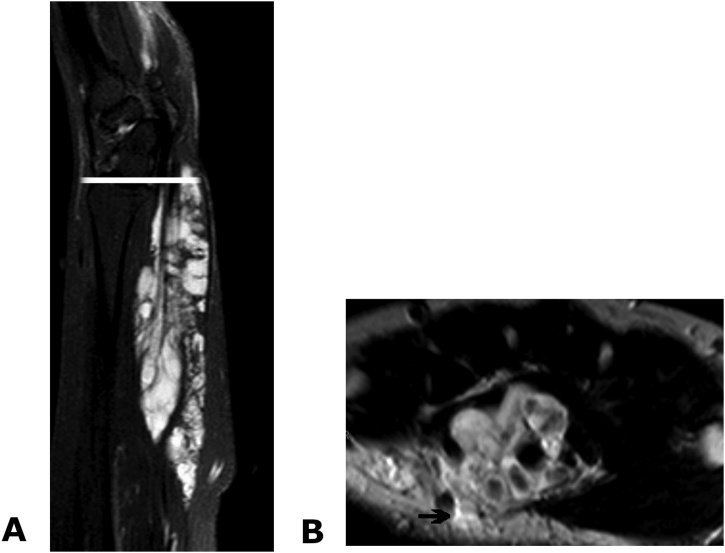
MRI showing expansion of the venous malformation in the carpal tunnel, resulting in compression of the median nerve (arrow). Left, sagittal view; right, axial view at the level of the bar in the left image.

Imaging findings showed no abnormalities on plain X-rays. MRI revealed hyperintensity on T2-weighted imaging and suggested that the vein had expanded from the proximal forearm to the palm (Fig. 2). The size of the lesion was 16 × 2.2 × 3.3 cm. The median nerve was compressed by the vascular malformation invading the carpal tunnel.

**Fig. 3:**
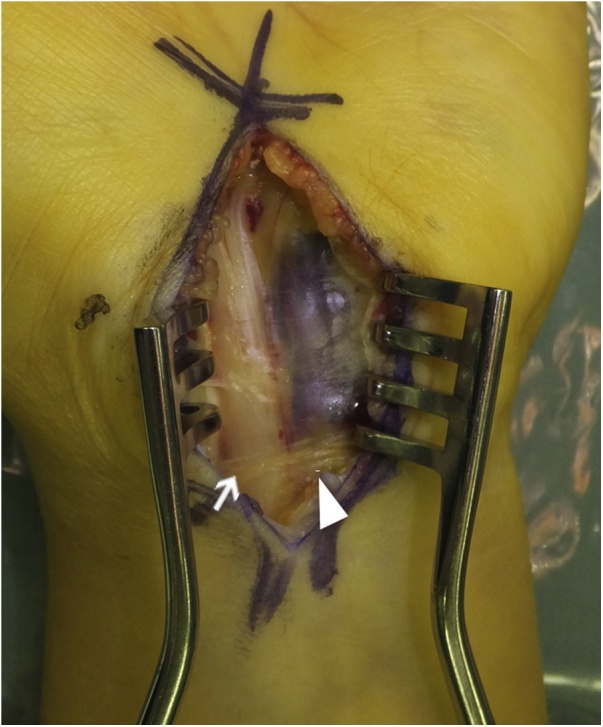
Intraoperative photograph taken with the carpal tunnel held open. showing the swollen vein (arrowhead) compressing the median nerve (arrow).

Pregabalin was given for pain, but there was no improvement. Resection of the vascular malformation was considered to be invasive and to have a risk of incomplete resection. Therefore, carpal tunnel release was performed as an alternative treatment. A skin incision was made from the distal forearm to the palm, and the flexor retinaculum was opened. The veins were present on the ulnar side of the median nerve (Fig. 3) and were not touched during the procedure to avoid damage. Postoperatively, pain gradually diminished. One year after the operation, numbness of the finger disappeared and the patient was able to return to work. She has had no recurrence of symptoms due to the vascular malformation to date.

## 3 Discussion

This case shows that vascular malformation can present as a nerve compression syndrome in middle age, after appearing in childhood. A less invasive operative treatment of carpal tunnel release was effective for reducing pain. Vascular anomalies are divided into malformations and tumors such as hemangioma, which both occur in infancy and childhood. Hemangiomas display rapid early growth, and resection may be the only treatment that can control growth [1]. Several reports of hemangioma of the median nerve have shown successful removal [[4], [5], [6], [7], [8]]. In contrast, vascular malformations tend to grow slowly and may involve local tissue and cause morbidity in adult age. A more conservative approach is required for treatment of these lesions [1].

The presence of a tumor or a tumor-like lesion should be considered as a possible, but rare, cause of nerve compression syndrome. A review of 541 cases of nerve compression syndrome found only three caused by vascular tumors, including a case with an intraneural invasive vascular tumor that could not be removed, resulting in unchanged paresthesia [9]. In a review of 128 cases of carpal tunnel syndrome, seven had space-occupying lesions, including occult ganglions, but none had vascular tumors [2].

In our case, finger numbness and pain were caused by median nerve compression at the carpal tunnel. The lesion itself was not painful. MRI showed relatively large expansion of the lesion from the proximal forearm to the palm through the carpal tunnel. In the carpal tunnel, the median nerve was separate from the venous structure, and the condition was diagnosed as an extraneural type. Resection of the lesion may have resulted in incomplete excision with subsequent persistent expansion and uncontrollable hemorrhage. Therefore, we performed carpal tunnel release, which is less invasive and does not have these risks, and this procedure improved the symptoms. The lesion remained untouched, and long-term follow-up will be important to evaluate the need for additional interventions if there is further expansion.

The symptoms of an intraneural vascular malformation may not be improved by carpal tunnel release. Furthermore, surgical excision may be most effective when the lesion is limited to the hand and upper extremity. Conservative treatment such as sclerotherapy and embolization can also be considered if surgery is inadequate, but these procedures also have a risk of nerve injury or ischemia because of the agent and blood loss [1].

## 4 Conclusion

In this case, vascular malformation in middle age presented as pain due to expansion of the lesion from the proximal forearm to the palm through the carpal tunnel. Less invasive treatment of carpal tunnel release was effective for reducing pain, as an alternative to resection of the lesion.

## Declaration of Competing Interest

None of the authors have a conflict of interest.

## Funding

This study did not receive any external funding.

## Ethical approval

A case report does not require approval from the Ethics Committee in our hospital.

## Consent

Written informed consent was obtained from the patient for publication of this case report and accompanying images.

## Author contribution

Morisaki S and Fujiwara H wrote the manuscript and prepared the figures. Morisaki S was the main surgeon, and is a specialist in hand surgery. Tsuchida S and Oda R contributed to the conception and design of the study and critically revised the manuscript. All authors read and approved the final manuscript.

## Registration of research studies

Name of the registry: researchregistry5540.

## Guarantor

Shinsuke Morisaki.

## Provenance and peer review

Not commissioned, externally peer-reviewed.

## References

[1] Taghinia A.H., Upton J. (2018). Vascular anomalies. J. Hand Surg..

[2] Nakamichi K., Tachibana S. (1993). Unilateral carpal tunnel syndrome and space-occupying lesions. J. Hand Surg..

[3] Agha R.A., Borrelli M.R., Farwana R., Koshy K., Fowler A.J., Orgill D.P. (2018). The SCARE 2018 statement: updating consensus surgical CAse REport (SCARE) guidelines. Int. J. Surg..

[4] Coessens B., De Mey A., Lacotte B., Vandenbroeck D. (1991). Carpal tunnel syndrome due to an haemangioma of the median nerve in a 12-year-old child. Ann. Chir. Main Memb. Super..

[5] Gonzalez Porto S.A., Gonzalez Rodriguez A., Midon Miguez J. (2016). Intraneural venous malformations of the median nerve. Arch. Plast. Surg..

[6] Louis D.S., Fortin P.T. (1992). Perineural hemangiomas of the upper extremity: report of four cases. J. Hand Surg..

[7] Patel C.B., Tsai T.M., Kleinert H.E. (1986). Hemangioma of the median nerve: a report of two cases. J. Hand Surg..

[8] Peled I., Iosipovich Z., Rousso M., Wexler M.R. (1980). Hemangioma of the median nerve. J. Hand Surg..

[9] Martinez-Villen G., Badiola J., Alvarez-Alegret R., Mayayo E. (2014). Nerve compression syndromes of the hand and forearm associated with tumours of non-neural origin and tumour-like lesions. J. Plast. Reconstr. Aesthet. Surg..

